# In Response to Michael Wininger’s Commentary: Common Roadblocks for Biomaterials Metrologists

**DOI:** 10.3390/jfb7030020

**Published:** 2016-07-27

**Authors:** Andrew Naylor

**Affiliations:** School of Mechanical and Systems Engineering, Newcastle University, Newcastle upon Tyne, England NE1 7RU, UK; andrew.naylor@newcastle.ac.uk; Tel.: +44-758-020-7250

I welcome Wininger’s commentary [1], and I would like to take the opportunity to address some of the issues raised. I was the lead author on the study at the center of the commentary [2] and hence I decided to take the lead in formulating this reply, which covers aspects of the research conducted by myself and my co-authors. We previously used both average roughness (S_a_) and skewness (S_sk_) to evaluate the bearing surfaces of two designs of finger prostheses (both tested in vitro): pyrolytic-carbon on pyrolytic-carbon [3]; and cobalt-chromium on ultra-high-molecular-weight-polyethylene [4]. In the study at the center of Wininger’s commentary [2], we used both S_a_ and S_sk_, along with root mean square roughness (S_q_) and kurtosis (S_ku_), to evaluate the surfaces of unused pyrolytic-carbon prostheses.

## 1. So Why Did We Select These Parameters?

We were aware that similar topographical studies used S_a_ [5,6,7]; S_q_ [6,7]; and S_sk_ [6,7] to evaluate prostheses for a range of joints. We also found compelling evidence from an industrially focused study [8] to link surfaces with high S_sk_ values to improved distribution of lubricant. We deemed it prudent to include the latter in our analysis, despite the fact that we could not find any bio-medically focused studies that had used it previously. Furthermore, these parameters are considered standard measures for surface topography and are included in ISO 25178-2 [9]. We felt that this further justified our use of S_a_; S_q_; S_sk_; and S_ku_ to evaluate the surfaces in our recent study.

## 2. Key Points Raised by Wininger

### 2.1. Sample Size

**Figure 1:**
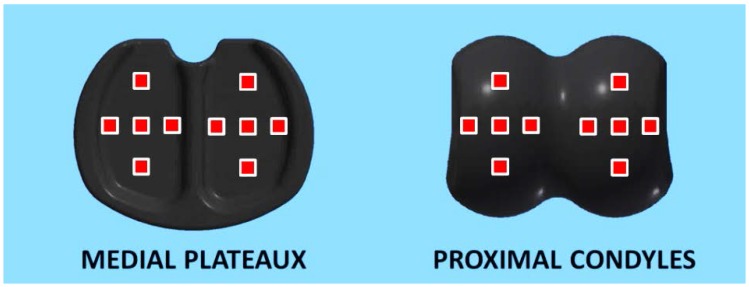
The locations on the prosthesis surfaces where the topographic plots were obtained.

Wininger raised the issue of sample size and makes two inaccurate assertions of our study. First he claims that we only evaluated “a single specimen per phalanx”; and then states “it should be strongly encouraged that studies based on a single specimen be withheld until additional samples can be collected and analyzed”. I do not want readers of Wininger’s commentary [1] to think that we only evaluated one sample and I urge the readers to refer to our study [2] before forming any biases. We were in fact able to secure two prosthesis pairs for each of the four nominal sizes produced by the manufacturer. For each of the eight prosthesis pairs we took ten topographical measurements from the proximal condyles; and a further ten from the medial plateaux. Figure 1 illustrates the sampling approach used to obtain the 160 measurements.

### 2.2. Roughness Parameters

Wininger has proposed a novel roughness parameter described as the spatial analogue to the *jerk*, a phenomenon commonly encountered in kinematics. I feel that Wininger’s goal to implement such a parameter is admirable, and I fully acknowledge that use of any one of the four previously mentioned parameters used in isolation cannot adequately describe a given surface. In our study we set out to measure these four parameters; we subsequently discussed which are the most appropriate; and which combinations work well together to describe surface phenomena. To paraphrase the findings of our study: we reported no need to measure both S_a_ and S_q_ as they are proportional to one another, with the latter returning only slightly higher values in magnitude; we reported that surfaces with a combination of negative S_sk_ and high S_ku_ values indicate the presence of sharp valleys, which could hold the key to improved lubricant distribution. To summarize our position, we are aware of the limitations of using just one roughness parameter and we are moving towards more rigorous methods of surface characterization.

**Figure 2:**
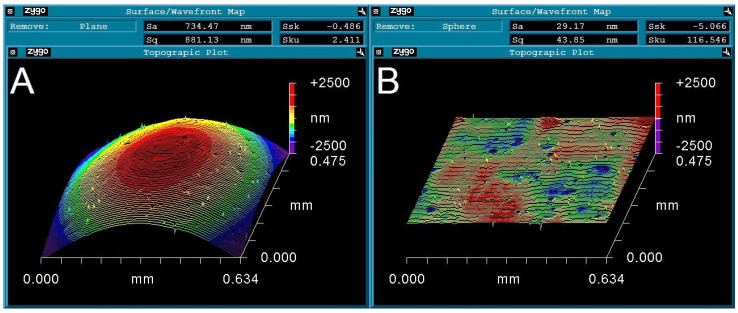
(**A**) Uncorrected topographic plot showing spherical profile; (**B**) Corrected topographic plot with flattened profile.

Wininger illustrated how the *jerk* parameter can be used to distinguish between two very different surface formations both with the same skewness. In this example one surface was smooth but inclined; the other was a random rearrangement of the heights showing a relatively level but rough surface. This example demonstrates the problems encountered by metrologists when dealing with profiles that are not flat. In our study we used the software MetroPro (Zygo Corporation, Middlefield, CT, USA), which has an in-built feature capable of accounting for spherical or cylindrical profiles. Take for instance a spherical surface that has not yet been corrected (Figure 2A). The high apex and the low slopes are merely read as relative heights and are subsequently used to calculate false roughness parameters. Once the spherical profile has been negated (Figure 2B) it is possible to quantify the true topography of the surface. Note the difference in the S_a_; S_q_; S_sk_ and S_ku_ values presented in Figure 2A,B.

I thank Wininger for acknowledging that we have adhered to established practices. I accept that the standards governing surface roughness measurement may not be perfect, my co-authors and I will consider Wininger’s useful comments prior to conducting out next topographical study. I hope that this response sheds more light on the rationale used for selecting the parameters that we did; and provides further explanation of the methods that we used.

## References

[1] Wininger M. (2016). Common roadblocks for biomaterials metrologists. J. Funct. Biomater..

[2] Naylor A., Talwalkar S., Trail I., Joyce T. (2016). Evaluating the surface topography of pyrolytic carbon finger prostheses through measurement of various roughness parameters. J. Funct. Biomater..

[3] Naylor A., Bone M.C., Unsworth A., Talwalkar S.C., Trail I.A., Joyce T.J. (2015). In vitro wear testing of the PyroCarbon proximal interphalangeal joint replacement: Five million cycles of flexion and extension. Proc. Inst. Mech. Eng. Part H.

[4] Naylor A., Talwalkar S.C., Trail I.A., Joyce T.J. (2015). In vitro wear testing of a CoCr-UHMWPE finger prosthesis with hydroxyapatite coated CoCr Stems. Lubricants.

[5] Bone M.C., Giddins G., Joyce T.J. (2014). An analysis of explanted pyrolytic carbon prostheses. J. Hand Surg. (Eur. Vol.).

[6] Scholes S.C., Kennard E., Gangadharan R., Weir D., Holland J., Deehan D., Joyce T.J. (2013). Topographical analysis of the femoral components of ex vivo total knee replacements. J. Mater. Sci. Mater. Med..

[7] Hall R.M., Siney P., Unsworth A., Wroblewski B.M. (1997). The effect of surface topography of retrieved femoral heads on the wear of UHMWPE sockets. Med. Eng. Phys..

[8] Sedlaček M., Podgornik B., Vižintin J. (2012). Correlation between standard roughness parameters skewness and kurtosis and tribological behaviour of contact surfaces. Tribol. Int..

[9] British Standards Institute (2012). Geometric Product Specification, Surface Texture (Areal). Part 2: Terms, Definitions and Surface Texture Parameters.

